# Editorial: Biomarkers in neurology, volume II

**DOI:** 10.3389/fneur.2023.1244536

**Published:** 2023-07-12

**Authors:** Wael M. Y. Mohamed, Firas Kobeissy, Stefania Mondello

**Affiliations:** ^1^Basic Medical Science Department, Kulliyyah of Medicine, International Islamic University Malaysia, Kuantan, Pahang, Malaysia; ^2^Department of Neurobiology, Center for Neurotrauma, Multiomics & Biomarkers (CNMB), Morehouse School of Medicine, Neuroscience Institute, Atlanta, GA, United States; ^3^Department of Biomedical and Dental Sciences and Morphofunctional Imaging, University of Messina, Messina, Italy

**Keywords:** biomarkers, neurological disorders, TBI, mTBI, HMGB1

Early diagnosis of most neurological disorders is considered a mainstay for early and successful management. The difficult accessibility to brain tissue represents a major problem in the diagnosis of brain diseases. Moreover, the evaluation of disease progression and predicting prognosis is hampered by the lack of sensitive and specific diagnostic tools. Biomarkers' definition exceeds the conventional understanding of chemical changes that accompany the disease process. Nowadays, we have radiological biomarkers, genetic biomarkers, and even microbial biomarkers (microbiomarkers). In some cases, early diagnostic clinical tests are considered biomarkers. As such, research in the field of biomarkers development is multidisciplinary, involving biochemists, geneticists, neurologists, radiologists, and more.

The demand for biomarkers, in particular those that may be used to stratify for therapy eligibility and to assess treatment success, is growing as new therapeutic options emerge. The function of a biomarker determines how it should be categorized. In the United States, the Food and Drug Administration (FDA) has established a task force that has identified seven broad classes of biomarkers; (1) first, there are diagnostic biomarkers, which aid in the detection or confirmation of a disease's presence or the identification of individuals with a subtype of the disease; (2) second, there are prognostic biomarkers, which determine the likelihood of a clinical event or disease progression in a patient with the disease; (3) third, there are predictive biomarkers, which determine which patients are more likely to benefit from a given medical product. (4) Fourth, response bio-markers, which show that a biological response has occurred in an individual exposed to medical treatment (for example, pharma-codynamic biomarkers, which measure the biological activity of the medical product, not necessarily concluding clinical outcome), (5) fifth, monitoring biomarkers, which allow for repeated assessment of the status of a disease or the effect of medical treatment, (6) safety bio-markers, which indicate toxicity of medical treatment, and finally, (7) risk biomarkers (1). Although there has been a lot of research on potential biomarkers, there is still a long way to go. The identification of several multimodal potential biomarkers for diagnostic, prognostic, and predictive purposes has not (yet) resulted in the emergence of a single, definitive marker. This is why the purpose of this Research Topic was to initiate a discussion on strategies for creating, validating, and accrediting biomarkers for brain injury. Given the wide variety of possible biomarkers, this concept is accessible to a wide range of researchers. Our project's ultimate goal was to identify a sensitive and specific biomarker for brain illness that is also simple, accessible, and inexpensive. Also, before a biomarker is used in clinical settings, we'd want to draw your attention to the importance of validating it. Quite simply, a paradigm change in the treatment of brain illness might result from the discovery of a reliable biomarker.

In this Research Topic, Wang et al. conducted a study aimed to assess the clinical utility of serum ANXA7 as a predictor of severity, early neurological deterioration (END), and prognosis after intracerebral hemorrhage (ICH). A total of 126 people with ICH and 126 people without ICH served as controls for this study. The National Institutes of Health Stroke Scale (NIHSS) was used to quantify the severity of stroke-related symptoms. The ABC/2 approach was used to calculate the volume of the ICH lesion. Patients had considerably greater blood ANXA7 levels compared to controls, and these levels were shown to be independently linked with the NIHSS score. Therefore, serum ANXA7 may be a valuable blood-derived biomarker for evaluating ICH severity, END, and prognosis.

Similarly, Yan et al. proposed that the protein Nrf2 (nuclear factor erythroid 2-related factor 2) may have a neuroprotective function in the body naturally. They aimed to determine whether or not serum Nrf2 is a useful prognostic indicator of severe Traumatic Brain Injury (TBI). Serum Nrf2 levels were measured in 105 healthy individuals and 105 people with traumatic brain injury as part of prospective cohort research. Using multivariate analysis, they were able to determine its associations with trauma severity and 180-day overall survival, death, and poor prognosis (extended Glasgow Outcome Scale score 1–4). Patients' serum Nrf2 levels were significantly higher than those of healthy controls. The substantial predictive impact of serum Nrf2 in mTBI (mild Traumatic Brain Injury) is therefore supported by the strong correlation between elevated serum Nrf2 levels and traumatic severity and outcome. In addition to pointing the way for future mTBI investigations, the study by Malik et al. underlines the lack of unanimity in the methodology of studies that assess inflammatory cytokines in the blood after mTBI.

CSF levels of leukocytes, chloride, glucose, aspartate aminotransferase, lactate dehydrogenase, adenosine deaminase, lactic acid, and protein were examined prior to ventriculoperitoneal shunt placement to determine their association with infection. When the cerebrospinal fluid glucose level is 2.8 mmol/L and the lactic acid level is >2.8 mmol/L, it is recommended to perform ventriculoperitoneal shunt after further improvement of cerebrospinal fluid indicators, otherwise, hasty operation will increase the postoperative infection rate in adults with hydrocephalus who do not have clinical manifestations of intracranial infection. Surgery with a ventriculoperitoneal shunt is associated with a high postoperative fever rate and a quick decline in body temperature. After 3 days post-op, the possibility of an intracranial infection should be explored if the patient still has a fever (Zhang J. et al.). Lei et al. looked at 125 people (onset 65) from the PUMCH dementia cohort, who were recruited systematically and divided into Alzheimer's disease (AD), non-AD dementia, and control groups. ELISA INNOTEST (Fujirebio, Ghent, Belgium) was used to determine amyloid- 42 (A42), total tau (t-tau), and phosphorylated tau (p-tau) levels. The differences between the groups are analyzed using either the students' *t*-test or a non-parametric test. To demonstrate the diagnostic efficacy of markers, the area under the receiver operating characteristic (ROC) curve (AUC ROC) was developed. To improve the diagnostic capability of a panel of indicators, a diagnostic model is established using logistic regression. Their findings provide credence to the use of biomarkers and established cutoff values in the diagnosis of AD in its earliest stages. They demonstrated that using ratios of t-tau to A42 and p-tau to A42 is more sensitive than using A42 levels alone and that adding biomarkers may further increase diagnostic accuracy. Similarly, cerebrospinal fluid vessel disease (CSVD) is prevalent in the elderly. Recent research has shown a link between NGAL (neutrophil gelatinase-associated lipocalin) and CVD and CVA (Cerebrovascular Accident). Inflammation brought on by NGAL damages the vascular endothelium and contributes to the development of various illnesses. The researchers aimed to find out whether CSVD patients' blood NGAL levels were a reliable indicator of how severely their illness will progress (Yang et al.).

Multiple genetic and environmental variables have been linked to the development of epilepsy, making it one of the most common neurological illnesses. Recent studies of the human scalp and cerebral EEG have shown novel markers for high-frequency oscillations (HFOs) between 80 and 500 Hz. HFOs may be produced in the brains of newborns. Newborns at risk for developing epilepsy may have a detectable biomarker in HFO. Preliminary findings from Kuhnke et al. imply that HFOs are more common in infants with abnormal background activity. To determine whether HFO incidence is related to seizure production and if this could predict the onset of epilepsy, larger data sets are required. As a new biomarker for epilepsy diagnosis, prognosis, and therapy, the aberrant expression patterns of miR-146a in various kinds and stages of epilepsy and its putative molecular control mechanism are summarized by Mao S. et al.. Similarly, the potential of HMGB1 (High-Mobility Group Box 1) as a biomarker for neurodegenerative illnesses such as Parkinson's disease, stroke, traumatic brain injury, epilepsy, autism, depression, multiple sclerosis, and amyotrophic lateral sclerosis was discussed by Mao D. et al.. The underlying biological pathways and clinical translation need more study in the future. The immune system causes the neuropathy known as Guillain-Barre syndrome (GBS). This suggests that the neutrophil-lymphocyte ratio (NLR) might serve as a diagnostic for its effectiveness. To summarize the evidence for NLR as a possible biomarker for GBS, Cabanillas-Lazo et al., did a systematic review and meta-analysis.

## Conclusion

In the current Research Topic, we highlighted the importance of the development of new biomarkers for early diagnosis of many neurological disorders. We are at a pivotal juncture in human history, one that may forever alter our culture's approach to the treatment of neurological illnesses. Developing effective policies for preventing, assessing, and treating many neurological disorders, as well as allocating responsibility when they occur, is hindered by our current inability to use sufficiently objective tests for diagnosis, prognosis, and monitoring of neurological disorders. While the objective symptoms of certain neurological disorders are being established, the great majority are not yet suitable for use in a clinical context where they may influence diagnosis, prognosis, and therapy. However, despite their potential, most of them are not ready for implementation in established legal or policy frameworks. There is a risk of exploitation of the legal system for unjust gain if blood biomarkers are not adequately standardized for their correct and reliable application in both the therapeutic and legal spheres. In all of this, courts will have the difficult issue of deciding what scientific evidence is admissible. The ultimate goal of biomarker development is to enhance clinical treatment for persons who have suffered from neurological illnesses, to create legislation that is consistent and well-informed concerning certain neurological diseases like TBI, and to provide more accurate and fair outcomes in litigation involving neurological disorders.

## Author contributions

All authors listed have made a substantial, direct, and intellectual contribution to the work and approved it for publication.
